# 

**Published:** 1848-01

**Authors:** 


### CONTENTS

OF THE

### DENTAL REGISTER OF THE WEST.

### JANUARY NO., 1848.

Art. 1, Letter from the St. Louis Editor, ........ Page 57.
Art. 2, The Lathe, by A. M. Leslie, D. D. S., ........ 63.
Art. 3, Vitality of the Teeth, by Wm. B. Ross, D. D. S., ........ 70.
Art. 4, Filling Teeth, by E. Taylor, M, D., D. D. S., ........ 73.
Art. 5, On Whole Setts of Teeth, by W. E. Ide, M. D., ........ 78.
Art. 6, Facts for the People, by H. Crane, D. D. S., ........ 82.
Art. 7, Chloroform, as a New Anæsthetic Agent, ........ 85.
Art. 8, Review of the Proceedings of the A, S. of D. S., by E. Taylor, M. D., D. D. S., ........ 93.
Art. 9, Contour of the Face, continued, by J. Allen, D. D. S., ........ 103.
Art. 10, Letter from Dr. Smith, ........ 105,
Art. 11, Brevities, by E. Taylor, D. D. S., ........ 106.
Art. 12, Chloroform, by Cin. Ed., ........ 107.
Art. 13, The 2d No of the Register, by Cin. Ed., ........ 108.
Art. 14, To the Members of the Mississippi Valley Association of Dental Surgeons, by Cin. Ed., ........ 109.
Art. 15, Repairing Instruments, by St. L. Ed., ........ 109.
Art. 16, Cleaning Files, by St. L, Ed., ........ 110.
Art. 17, Cutlery, by St. L, Ed., ........ 110.
Art. 18, Annealing Gold Foil, by St. L. Ed., ........ 111.
Art. 19, Manufacture of Gold Foil in St. Louis, Mo., by St. L. Ed., ........ 111.
Art. 20, Third Edition of Dr. Harris' Work on Dentistry, by Cin. Ed., ........ 111.
Art. 21, Typographical Errors, by Cin. Ed., ........ 111.
Art. 22, Cincinnati and St. Louis, by Cin. Ed., ........ 112.
Art. 23, Exchange List, by Cin. Ed., ........ 112.
Art. 24, New Method of Operating, by Cin. Ed., ........ 112.

